# Comparative Evaluation of Permissiveness to Dengue Virus Serotype 2 Infection in Primary Rodent Macrophages 

**DOI:** 10.1155/2012/950303

**Published:** 2012-03-08

**Authors:** Jeanette Prada-Arismendy, Verónica Rincón, Jaime E. Castellanos

**Affiliations:** Instituto de Virología, Universidad El Bosque, Carrera 7B Bis No. 132-11, Bogotá, Colombia

## Abstract

Infection with dengue virus presents a broad clinical spectrum, which can range from asymptomatic cases to severe cases that are characterised by haemorrhagic syndrome and/or shock. The reason for such variability remains unknown. This work evaluated the *in vitro* permissiveness of mouse, rat, hamster and guinea pig macrophages to infection by dengue virus 2 (DENV2). The results established that macrophages derived from the BALB/c mouse strain showed higher permissiveness to DENV2 infection than macrophages from other rodent species, although all rodent species studied had the C820T mutation in the oligoadenylate synthetase 1b gene, indicating no relationship to the different *in vitro* susceptibilities of mouse cells at this locus. Other molecular mechanisms related to flavivirus susceptibility remain to be explored.

## 1. Introduction

Infection with dengue virus (DENV) causes dengue and severe dengue (formerly dengue fever and dengue haemorrhagic fever). DENV belongs to the *Flaviviridae* family, flavivirus genus. DENV is an enveloped virus, and its genome consists of a positive polarity, single-stranded RNA of around 11 kb that encodes ten proteins. DENV can replicate in several types of cells, including dendritic cells, B and T lymphocytes, endothelial cells, hepatocytes, and neuronal cells. However, monocytes/macrophages are the primary target during *in vivo* infection [1]. Viral entry into these cells enables the virus to spread to different tissues and induces the presentation HLA molecule-associated viral antigens. The presentation of viral antigens by macrophages to memory T cells induces T cell activation and, consequently, the proliferation and production of cytokines such as TNF-*α*, IFN, and IL-2. This set of cytokines and chemokines induces endothelial dysfunction and plasma leakage, both of which are characteristic of more severe manifestations of the disease [2].

Several studies have addressed the relationship between human genes and the susceptibility to dengue virus infection. For example, the HLA-A*0203 allele correlated with dengue fever (DF) in Thai children, while HLA-A*0207 correlated with dengue haemorrhagic fever (DHF). Moreover, the alleles HLA-B44, B62, B76, and B77 confer protection against DHF in secondary infections [3]. Other polymorphisms associated with DHF have been found in the genes for DC-SIGN1, TNF-*α*, Fc-*γ* receptors, vitamin D receptors, and mannose binding lectin [4, 5]. In addition to these host factors, there is evidence that DENV serotypes 2 and 3 cause DHF more frequently [6], supporting the belief that both host and virus factors can affect the clinical outcome of patients.

Recent studies have identified the oligoadenylate synthetase 1b (Oas1b) gene as being responsible for susceptibility/resistance to West Nile Virus (WNV) infection in mice, and it is therefore a potential candidate gene for the *Flv* locus [7, 8]. These seminal works found a non-sense mutation (C820T) in the Oas1b gene, exon 4, that was linked to susceptibility to WNV infection. This mutation replaces an arginine with a stop codon, producing a truncated protein lacking the C-terminal domain. Knock-in of a normal Oas1b allele induced resistance to Yellow Fever Virus infection in a susceptible mouse strain, which was comparable to the resistance observed in resistant congenic mice [9]. These results led to the conclusion that the Oas1b gene conferred resistance against flavivirus infection. However, a neuronal cell line expressing the complete form of the Oas1b protein showed only a slight reduction in virus yield and was not significantly different from the same neuronal cell line expressing the truncated form [10]. Likewise, transfecting cDNA from the complete Oas1b gene into susceptible embryonic fibroblasts did not induce a complete reversion to the resistant phenotype [11]. Together, these results suggest that murine resistance to Flavivirus infection is not completely understood *in vitro* and that other cellular and animal models should be studied. This work evaluated the permissiveness to DENV infection and evaluated the presence of the C820T polymorphism in the Oas1b gene obtained from different rodent species, including three strains of mouse, rat, hamster, and guinea pig.

## 2. Materials and Methods

### 2.1. Animals and Cells

Experiments with animals were approved by the Universidad El Bosque's Ethics Committee following the national legislation. Different 6–8-week-old rodent species were used and were obtained from the Colombian National Institute of Health's animal facility. The animals used were BALB/c inbred mice (*Mus musculus*), NIH Swiss outbred mice, CD1 (ICR) Swiss outbred mice, Syrian hamsters (*Mesocricetus auratus*), Wistar rats (*Rattus norvegicus*), and Hartley strain guinea pigs (*Cavia porcellus*). Two millilitres of 1.5% carboxymethylcellulose was injected intraperitoneally into two animals from each species and this was done twice in different days. After 48 hours, the animals were euthanized, and a peritoneal wash was made with RPMI 1640 medium plus 10% foetal bovine serum, 100 U/mL penicillin, 10 *μ*g/mL streptomycin, and 2,5 *μ*g/mL amphotericin (complete medium-CM). The medium was collected and centrifuged, and the cell pellet was then suspended in CM. Then, 200,000 cells were seeded into 12-well culture dishes and 10,000 cells were seeded in 24-well culture dishes using round glass coverslips. Nonadherent cells were removed 48 hours later, and the culture of adherent cells continued for a further 5 days to allow for cytokine clearance. Adherent cells were considered to be peritoneal macrophages, as previously described [12]. The cells were seeded in duplicate from each animal used.

### 2.2. Virus and Infection

The virus was obtained by infecting C6/36 HT cells with a DENV serotype 2 (COL-789) isolate donated by Dr. Jairo Méndez from the Colombian Institute of Health's Virology Group and titrated on LLCMK-2 cells. The virus was placed in contact with the macrophages for 1 hour at 37°C at a multiplicity of infection (MOI) of 1 and 0.1; it was then removed, fresh medium was added, and the cells were kept for 24, 48, and 72 hours, at which time they were processed.

### 2.3. Plasmid Construction and qPCR

A capsid gene fragment amplified from DENV2 RNA was used to construct a plasmid as published previously [13], and a dilution having 10^10^ copies/*μ*L was prepared. RNA was extracted from infected and uninfected macrophage supernatants and monolayers using Trizol LS and Trizol reagents, respectively, (Invitrogen) and reverse-transcribed using the M-MLV enzyme and random primers. The SYBR Green and the GeneAmp 5700 sequence detection system (Perkin-Elmer Corporation) were used for the real-time PCR assay. A DENV capsid gene fragment was amplified using DV2C-L and DV2C-R primers. Samples lacking cDNA were used as negative controls, and cDNA obtained from viral inoculums was used as positive controls. The data obtained from the monolayers were analysed using the relative quantification method based on the assumption of ideal amplification efficiency with a doubling product every cycle, followed by twofold changes in fluorescence intensity (which could be calculated using the 2^−ΔΔCT^ formula) and *β*-actin as the housekeeping gene [14]. Absolute quantification was used for analysing the data obtained from the supernatants by producing a six-point standard curve (10^7^–10^2^ copies/*μ*L) from the pDV2core plasmid (10^7^–10^2^ molecules) that was obtained as previously described [13]. This curve was simultaneously processed with the samples and evaluated 24, 48, and 72 hours p.i. Data were analysed using ANOVA and a Least Significant Difference *post hoc* test and *P* < 0.05 was considered significant. Two independent experiments were done, and two animals were used from each species in each one. Independent duplicates of cells were seeded for each animal (*n* = 8); each sample was processed by duplicate in qPCR experiments.

### 2.4. Immunocytochemistry and TUNEL Assay

Infected and noninfected cells were fixed with 4% paraformaldehyde for the same defined periods and then permeabilised with 0.1% Triton X-100. Infected cells were detected by using monoclonal antiflavivirus antibody (Chemicon, MAB8744). Biotinylated anti-mouse IgG antibody was used as the secondary antibody and detected with peroxidase-coupled streptavidin, and 0.05% diaminobenzidine and 0.01% H_2_O_2_ were used as the developing reagents. Other cultures were incubated with biotinylated dUTP and the TdT enzyme for detecting DNA fragmentation (as an apoptotic indicator); after being washed, samples were incubated with Cy3-coupled streptavidin. The cultures were visualised and counted in a Wild Leitz GmBH fluorescence microscope. One-way ANOVA was done, and when the overall ANOVA resulted in a *P* value less than 0.05, Least Significant Difference (LSD) test was carried out for comparing infected cell counts and values from viral RNA quantification.

### 2.5. Obtaining and Sequencing DNA

Brain DNA was obtained from each rodent species using phenol/chloroform extraction and precipitated with sodium acetate and ethanol. This DNA was used to amplify an Oas1b gene exon 4 fragment using OAS-1bR: 5′-CTG GGA GTA TGG GAG TCG AG-3′ and OAS-1bL: 5′-GCT GTT GGT GCA GGT ATT CA-3′ primers, which amplify the gene region between nucleotides 771–925. The PCR product was purified and sequenced.

## 3. Results

### 3.1. Dengue Virus Replication Efficiency among Different Rodent Macrophages

**Figure 1:**
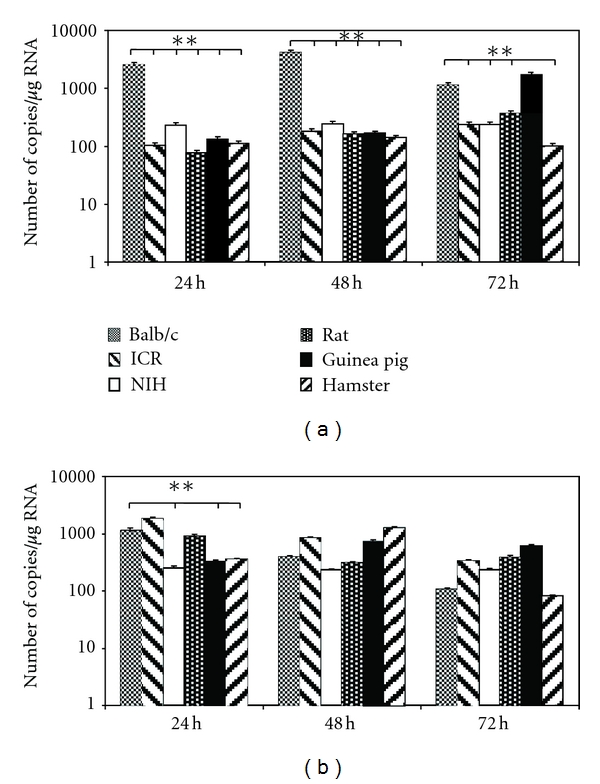
qRT-PCR to obtain absolute quantification of viral copies yielded to supernatants from different rodent species' peritoneal macrophages cultures. (a) Cells infected at a MOI 0,1 and (b) MOI 1. **P < 0.01.

The number of viral copies released by the macrophages into the supernatant was quantified by real-time PCR analysis of the RNA obtained from the supernatant of cells infected for 24, 48, and 72 hours at two different MOIs.  Figure 1(a) shows that the viral copy number obtained from BALB/c-derived macrophage supernatants infected with DENV at an MOI of 0,1 in all post-infection (p.i.) times was 4–25 times larger than that obtained from NIH- and ICR-derived mouse macrophages. The differences in the number of viral copies were significant at 24 and 48 hours p.i. (*P* < 0.01), while the number of viral copies obtained in BALB/c-derived macrophages was similar to that obtained in NIH- and ICR-derived mouse macrophages at 72 hours. Similar findings were established in macrophages from taxonomically different rodent species, such as rats, hamsters, and guinea pigs, where highly significant differences were found (*P* < 0.01).

When the macrophages were infected at an MOI of 1 (Figure 1(b)), significant differences were observed 24 hours p.i. when BALB/c donors were compared to NIH mice, hamsters, and guinea pigs (*P* < 0.01). No difference in the viral copy number were found in the supernatants at 48 and 72 hours p.i.

**Figure 2:**
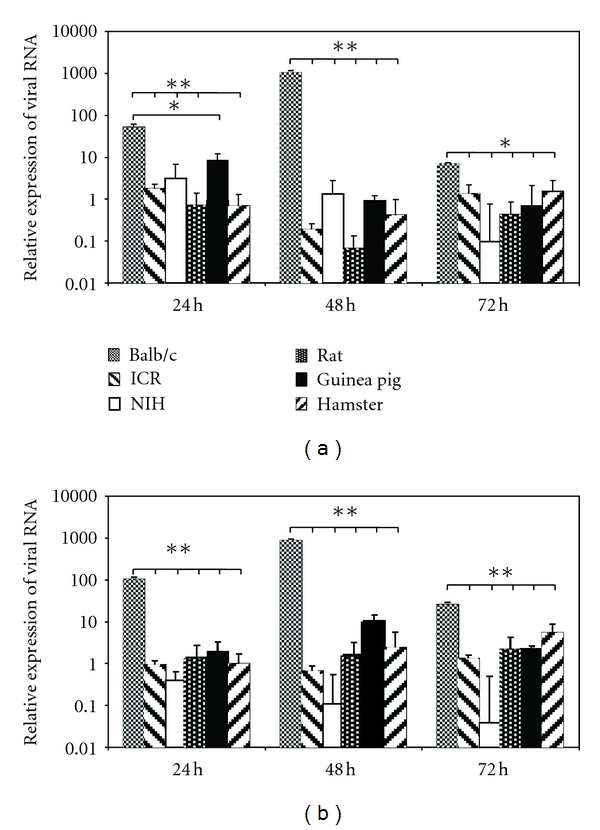
qRT-PCR to obtain relative quantification of viral copies obtained from monolayers from different rodents' peritoneal macrophages cultures. The fold change of viral RNA was calculated from DENV-infected macrophage monolayers, normalising the data obtained against noninfected control and against *β*-actin at 0,1 MOI (a) and at 1 MOI (b) and evaluated 24, 48, and 72 hours p.i. *P < 0.05, **P < 0.01.

A larger number of viral RNA copies were found in BALB/c-derived macrophage supernatants from cells infected with an MOI of 0.1 than with an MOI of 1 (Figure 1(a) versus Figure 1(b)). There were no differences among viral copies when the infection was carried out at an MOI of 0.1 or 1 in the other animals (*P* > 0.05). Relative RNA viral quantitation was carried out from macrophage monolayers and showed that the amount of virus found in cells was proportional to the amount of virus released to the supernatant. The relative viral RNA quantity was significantly larger in BALB/c macrophages, from 4-fold (BALB/c versus hamster) to 5400-fold (BALB/c versus ICR) higher at an MOI of 0,1, and from 4-fold (BALB/c versus hamster) to 8100-fold (BALB/c versus ICR) at an MOI of 1 (*P* < 0.05, see Figure 2). Differences in relative expression levels were much more marked than the differences found in the absolute quantification of the supernatants. The data obtained from the other rodent species did not reveal any significant differences when comparing different MOIs, nor when comparing the three p.i times. With the exception of the lower relative viral RNA levels found in NIH mouse macrophages, the levels of viral RNA tended to decrease as the p.i. time increased (Figure 2).

### 3.2. Infected and Apoptotic Macrophages from Different Rodents

**Figure 3:**
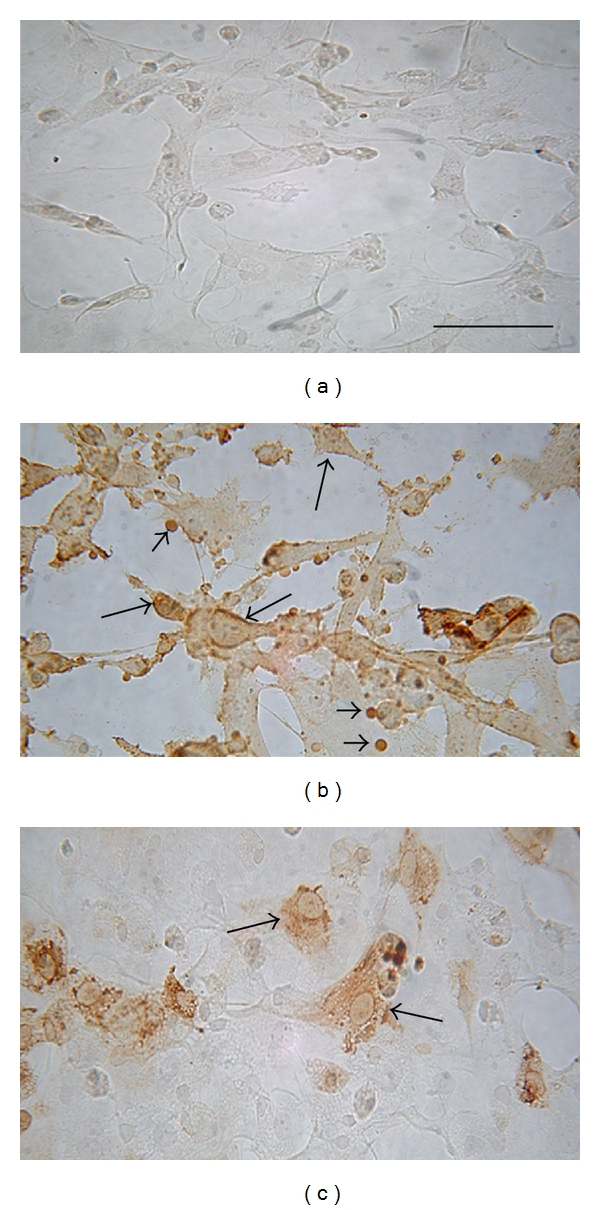
Immunocytochemistry for DENV2-infected or noninfected macrophages. (a) Noninfected macrophage control. (b) Macrophages from DENV-infected BALB/c mice at 1 MOI, 72 hours p.i. Observe the staining in almost all cells (arrows). Note the extracellular vesicles immunoreactive to viral antigen (arrow heads). (c) Hamster macrophages DENV-infected at MOI 1, 72 hours p.i. The stained cells show an intracytoplasmatic viral antigen distribution; the other animals showed a similar labelling pattern. The bars correspond to 40 *μ*m.

Immunocytochemistry was used to detect viral antigens in macrophages from the six different rodent species studied. As previously described [15], specific immunoreactivity was observed that corresponded to viral antigens in the infected cell cytoplasm (having punctiform characteristics) in the perinuclear region. A significantly larger number of infected macrophages was found in BALB/c-derived macrophages compared with other rodent macrophage cultures (Figure 3(c)). Approximately 43% of the macrophages from BALB/c mice inoculated with an MOI of 1 showed evidence of infection 24 hours p.i., increasing to 100% at 48 hours p.i.; an 81% infection of macrophages was reached at an MOI of 0.1, but only at 72 hours p.i. infection percentages were much lower in cells from the other animals (see Table 1). A large number of positively stained extracellular vesicles were also observed in BALB/c-derived macrophages (Figure 3(b)), but they were not observed in macrophages from other species (Figure 3(c)). Macrophages inoculated with DENV at an MOI of 1 presented a high percentage of DNA fragmentation, as demonstrated by TUNEL. High apoptotic percentages were found from 24 hours p.i. (88%) onwards and remained high up to 48 and 72 hours p.i. (91 and 99%, resp.). In infected macrophages from rodent species other than BALB/c, the percentage of TUNEL-positive cells was similar to that found in control cells that had not been infected with DENV (Table 1). An interesting finding was that the apoptotic percentages in macrophages from BALB/c mice inoculated with DENV at an MOI of 0,1 did not correlate with the percentages for DENV-positive cells. While 79% of the cells had become infected 48 hours after-infection, only 8% were apoptotic; the same phenomena occurred at 72 hours (81% versus 5%).

### 3.3. Presence of the C820T Mutation

Analysing the sequences from the Oas1b gene exon 4 fragment obtained from BALB/c mouse genomic DNA revealed that they were consistent with those reported previously [7, 8]. The TGA stop codon mutation inserts a premature translation stop in the Oas1b gene in these mice. Interestingly, the other rodent species analysed also had the C820T mutation, which leads to the translation of a truncated protein in all cases. The sequences from this Oas1b fragment from guinea pigs, hamsters, and NIH and ICR mice had not been previously identified and were reported to GenBank (*Cavia porcellus* accession number EF081023, *Rattus norvegicus* accession number EF081022, *Mus musculus* NIH strain accession number EF081021, *Mus musculus* ICR strain accession number EF081020, *Mesocricetus auratus* accession number EF081019, *Mus musculus *BALB/c strain accession number EF081018).

## 4. Discussion

Genetic markers for host susceptibility to infection could provide answers for questions concerning why not all individuals infected by DENV become ill and why they do not all have similar symptoms if they do become ill. The clinical manifestations of dengue virus infection range from undifferentiated fever to severe systemic compromise, encephalitis, and haemorrhagic syndrome. The reason why infection with DENV (and the flavivirus in general) causes clinical manifestations in only a small percentage of infected individuals remains unknown. However, it has been suggested that the host's genetic factors could be involved. The resistance of some mice strains to infection by some flavivirus has been described since the beginning of the twentieth century ([11] reviewed in [16]), and it was initially found that flavivirus resistance to infection was inherited as a dominant trait [17]. 

It is known that the monocyte/macrophage system is the primary *in vivo* cellular target for DENV, and the peritoneal macrophage model has also been used to study flavivirus resistance and susceptibility. In this work, it was found that macrophages from BALB/c mice had higher permissiveness to DENV2 infection using qPCR and immunocytochemistry. Sequence analysis of the Oas1b gene demonstrated that all four rodent species studied had the C820T mutation, leading to the supposition that the production of a truncated protein by this genotype does not affect DENV susceptibility. 

With the exception of BALB/c mice, all rodent strains and species of macrophages studied have a low capacity to support DENV2 replication because infection with the virus did not cause large amounts of RNA to assemble or be released as virions. This finding could contradict the suggestion of a protective mechanism involving viral packaging, as has been previously proposed [18]. The results found in NIH mouse-derived macrophages could suggest that these cells not only have a cellular mechanism that reduces permissiveness but also have a more efficient viral clearance process. Nevertheless, it is important to clear that these results are only relevant for dengue virus serotype 2; it is probable that different results could be found for other dengue serotypes.

Like other studies, we did find more viral copies in supernatants in any of the cultures that had been inoculated with an MOI of 0.1 relative to those with an MOI of 1. Brinton [19] suggested that this finding could be due to the presence of defective interfering (DI) particles found in greater amounts at a higher MOI. The production of DI particles could be facilitated by some mechanism in resistant cells, while highly susceptible cells will mainly produce infectious virus. In turn, this higher virus production leads to greater cell death in highly susceptible cells. The theory is supported by the work done by Espina et al. [20], who used human monocytes as their model. Despite the infected cell percentage in BALB/c-derived macrophages is similar with both MOIs, infection at an MOI of 1 induced a significantly higher number of TUNEL-positive cells. It seems that the infection of cells with a high MOI activates proapoptotic molecular routes as a mechanism to block viral replication and dissemination during viral infection, thereby explaining this finding. However, the virus could be activating anti-apoptotic routes at a low MOI and improving cell survival. It has been previously established that DENV induces apoptosis in different cell types (e.g., endothelial cells, hepatocytes, dendritic cells, monocytes, and neuroblastoma cells) [21]. The proapoptotic mechanisms that are related to DENV infection include apoptosis activation by the extrinsic route (via ligands such as TNF-*α* and Apo2/TRAIL) [22] and the intrinsic route (described in neuroblastoma cells having phospholipase A2 activation, superoxide anion production, cytochrome c release, followed by caspase 3 activation). A mechanism related to apoptosis has not been previously proposed in work studying resistance and susceptibility to flavivirus infection. The results of the present study displayed a clear relationship between lower susceptibility to infection and decreased apoptosis. This finding suggests that the control of apoptosis-inducing mechanisms may represent a macrophage protection strategy in addition to it control of viral replication, a phenomenon that deserves more investigation. It would also be necessary to consider in the study of these cells susceptibility to dengue virus infection, the recent findings of Kwan et al. [23], in which Monocyte-derived dermal macrophages were capable of internalizing live DENV but they displayed an inherent resistance to viral growth because virus particles accumulate into poorly acidified phagosomes, which prevents release of the nucleocapsid into the cytoplasm, and therefore its later replication. Together, these results suggest an additional level of complexity in the genetic control of flavivirus replication.

## 5. Conclusions

This work can conclude that BALB/c mice macrophages had higher permissiveness to DENV2 infection as it showed higher viral copies when measuring by using qPCR and immunocytochemistry. Besides, sequence analysis demonstrated that Balb/C, NIH, and ICR mice as well as rat, hamster, and guinea pig had the C820T mutation in Oas1b gene. It is needed to carry out more studies to clarify which is the molecular pathway involved in this higher permissiveness and if it is related to some apoptotic pathway.

## Figures and Tables

**Table 1:** Percentage of infected and positive TUNEL cultured macrophages from different rodents.

		0.1 MOI						1 MOI					
	MOCK	24 h		48 h		72 h		24 h		48 h		72 h	
	TUNEL	DENV+	TUNEL	DENV+	TUNEL	DENV+	TUNEL	DENV+	TUNEL	DENV+	TUNEL	DENV+	TUNEL
BALB/c	4.0	13.2	5.2	79.8	8.2	81.4	5.0	43.4	88.6	100	91	86.8	99.2
ICR	13.0	26.6	9.2	21.8	17.0	22.6	13.6	13.2	4.6	25.6	23.6	34.2	17.0
NIH	8.0	5.8	5.0	12.2	9.4	15.6	31.0	9.2	9.4	13.8	29.2	17.2	9.2
Rat	2.0	24.4	4.6	17.8	5.2	23	7.4	20.6	5.6	13.6	4.2	8.0	6.3
Guinea pig	5.2	5.2	3.2	12.4	8.6	24.4	4.6	5.2	6.0	17.8	8.0	29.6	3.8
Hamster	7.0	13.4	12.0	32.8	34.8	8.6	33.2	6.4	3.0	5.4	17.0	10	15.2

The data represent the percentage of DENV antigen positive over the total of cells counted. Note that only 43.4% of the cells were positive for viral antigen in BALB/c mouse macrophage infected with 1 MOI, 24 hours p.i., but almost all the cells presented DNA degradation. Two animals were used from each species; two groups of independent cells were seeded for each animal and two replicates were done for each of them in immunocytochemistry and TUNEL assays (*n* = 8).
